# Chromosomal Instability Drives Glioblastoma Heterogeneity and Therapeutic Opportunities

**DOI:** 10.1002/advs.202501015

**Published:** 2026-06-16

**Authors:** Amarnath Pal, Milan Patra, Rianita Mondal, Shubhasis Haldar

**Affiliations:** ^1^ Department of Chemical and Biological Sciences S. N. Bose National Centre for Basic Sciences Kolkata West Bengal India

**Keywords:** CIN, chromothripsis, heterogeneity, plasticity, therapeutic resistance

## Abstract

Glioblastoma, the most aggressive and lethal form of brain cancer, is defined by profound genomic instability, with Chromosomal Instability (CIN) playing a central role in driving tumor progression, therapy resistance, and poor prognosis. CIN is characterized by numerical and structural alterations, is driven by mechanisms such as mitotic errors, centrosome amplification, spindle assembly checkpoint dysfunction, and defective DNA repair pathways. These aberrations contribute to tumor heterogeneity, leading to the emergence of Glioblastoma Stem Cells (GSCs) with enhanced plasticity, therapy resistance, and metastatic capacity. Chromothripsis, frequently involves specific chromosomes and stems from micronuclei rapture, resulting in chromosomal rearrangement. The immune implications of CIN are also critical, with the Cyclic GMP–AMP Synthase–Stimulator of Interferon Genes (cGAS‐STING) pathway toggling between anti‐tumor immunity and immune evasion. Therapeutic strategies targeting CIN are explored, including inhibitors of centrosomal clustering, DNA damage response pathways, and spindle assembly components, as well as innovative approaches like Chimeric Antigen Receptor T (CAR‐T) cell therapies and nanoparticle‐based drug delivery systems. Advances in single‐cell sequencing provide transformative insights into CIN‐driven glioblastoma heterogeneity and therapeutic vulnerabilities. By integrating mechanistic understanding with translational strategies, this review underscores CIN as both a therapeutic challenge and an opportunity, charting a path toward improving glioblastoma treatment outcomes and patient survival.

## Graphical abstract

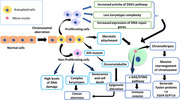

**Abbreviations**
2HG: D‐2‐hydroxyglutarate
ACVR1: Activin A Receptor Type I
ALDH: Aldehyde Dehydrogenase
ALT: Alternative Lengthening of Telomeres
AMEN: Atypia, Mitosis Index, Endothelial Cell Proliferation, and Necrosis
AP‐1: Activator Protein‐1
APC/C: Anaphase‐Promoting Complex/ Cyclosome
ATM: Ataxia‐telangiectasia Mutated
ATR–CHK1: Ataxia Telangiectasia and Rad3‐related Protein – Checkpoint Kinase 1
ATRX: Alpha‐Thalassemia/Mental Retardation Syndrome X‐linked
AURKA: Aurora Kinase A
AXL: AXL Receptor Tyrosine Kinase
BER: base‐excision Repair
BRCA1/2: Breast Cancer Gene 1/2
Bub1: Budding Uninhibited by Benzimidazoles 1
BubR1–Bub3: Budding Uninhibited by Benzimidazoles‐Related 1‐ Budding Uninhibited by Benzimidazoles 3
CA: Centrosome Amplification
CAA: Chromosomal Arm Aneuploidy
CAR‐T: Chimeric Antigen Receptor T
CBTRUS: Central Brain Tumor Registry of the United States
CCND2: Cyclin D2
Cdc20: Cell Division Cycle 20 Homologue
CDK4: Cyclin‐Dependent Kinase 4
CDKN2A/2B: Cyclin Dependent Kinase Inhibitor 2A / 2B
CEBP: CCAAT/Enhancer Binding Protein
CEP55: Centrosomal Protein of 55 kDa
cGAMP: Cyclic guanosine monophosphate–adenosine monophosphate
cGAS‐STING: Cyclic GMP–AMP Synthase–Stimulator of Interferon Genes
CII: Chromosomal Instability Index
CIN: Chromosomal Instability
c‐MYC: cellular myelocytomatosis oncogene
CNS: Central Nervous System
CNVs: Copy Number Variations
CPAP: Centrosomal P4.1 Associated Protein
CSCs: Cancer Stem Cells
DDR: DNA Damage Response
DSBs: double‐strand Breaks
E2F: E2 Promoter Binding Factor
Eg5: kinesin‐5 family Motor Protein
EGFR: Epidermal Growth Factor Receptors
EMT: Epithelial–mesenchymal Transition
ESCRT –III: Endosomal Sorting Complexes Required for Transport‐III
FGFR: Fibroblast Growth Factor Receptor
FISH: Fluorescence in Situ Hybridization
FOXM1: Forkhead Box M1 Signaling Axis
GBM: Glioblastoma Multiforme
G‐CIMP Phenotype: Glioma‐CpG Island Methylator Phenotype
GSCs: Glioblastoma Stem Cells
H3F3A: H3 Histone Family Member 3A
HER2: Human Epidermal Growth Factor Receptor 2
HGF/MET: Hepatocyte Growth Factor/ MET Proto‐Oncogene, Receptor Tyrosine Kinase
HR: Homologous Recombination
HSET: Human Kinesin Family Member C1
HsSAS‐6: Human Spindle Assembly Abnormal Protein 6 Homolog
IDH: Isocitrate Dehydrogenase
IFT: Intraflagellar Transport
IGF‐1: Insulin‐like Growth Factor
IKKα: Inhibitor of NF‐kβ Kinase α
IL13Rα2: Interleukin‐13 Receptor Subunit Alpha‐2
KDR: Kinase Insert Domain Receptor
K‐MT: kinetochore–microtubule
Knl1: Kinetochore Scaffold 1
KT: Kinetochore
LmnB1: Lamin‐B1
LOH: Loss of Heterogeneity
Mad1: Mitotic Arrest Deficient 1
MCC: Mitotic Checkpoint Complex
MDC1: Mediator of DNA Damage Checkpoint 1
MDM2/4: Mouse Double Minute 2 / 4
MDSCs: Myeloid‐Derived Suppressor Cells
MGMT: O6‐methylguanine‐DNA Methyltransferase
MMR: mismatch Repair
MN: Micronuclei
Mps1: Monopolar Spindle 1 Kinase
MRE11: Meiotic Recombination 11
MSH6: MutS Homolog 6
MT: Microtubules
mTOR: Mammalian target of Rapamycin
Ndc80: Nuclear Division Cycle 80
Nek2: NIMA Related Kinase 2
NF‐κB: Nuclear Factor Kappa B
NHEJ: Non‐homologous End Joining
NPM: Nucleophosmin
NuMA: Nuclear Mitotic Apparatus
NUP107: Nucleoporin 107
PARP: Poly (ADP‐ribose) Polymerase
PDGFR: Platelet‐Derived Growth Factor Receptor
PDGFRA: Platelet‐Derived Growth Factor Receptor Alpha
PI3K/AKT: Phosphatidylinositol 3‐Kinase/Protein Kinase B
PLK4: Polo‐Like Kinase 4
PPM1D: Protein Phosphatase, Mg2+/Mn2+ Dependent 1D
PTEN: Phosphatase and Tensin Homolog
PTPRD: Protein Tyrosine Phosphatase Receptor Type D
RAD51: Radiation Sensitive Protein 51
RAF–MEK–ERK: Rapidly Accelerated Fibrosarcoma‐ Mitogen‐activated Protein Kinase Kinase‐Extracellular‐signal‐regulated Kinase
RAS–RAF–MAPK: Ras/Rapidly Accelerated Fibrosarcoma/Mitogen‐Activated Protein Kinase
RB: Retinoblastoma Protein
RTK: Receptor Tyrosine Kinase
SAC: Spindle Assembly Checkpoint
SCF: Skp, Cullin, F‐box Containing Complex
SEPT14: Septin‐14
SOX2: SRY‐Box Transcription Factor 2
Sp1: Specificity Protein 1
SQSTM1: Sequestosome1
STAT3: Signal Transducer and Activator of Transcription 3
STIL: Stromal Tumor Infiltrating Lymphocytes
TAAs: Tumor‐Associated Antigens
TACC3: Transforming Acidic Coiled‐Coil Containing Protein 3
TCA: Tricarboxylic Cycle Acid
TCGA: The Cancer Genome Atlas
TERT: Telomerase Reverse Transcriptase
TET: Ten‐Eleven Translocation Enzyme
TMZ: Temozolomide
TP53: Tumor Protein 53
TWIST1: Twist family bHLH Transcription Factor 1
VEGF: Vascular Endothelial Growth Factor
VOPP1: Vesicular, Overexpressed in Cancer, Prosurvival Protein 1
WEE1: WEE1 G2 checkpoint Kinase
WHO: World Health Organization
YY2: Yin Yang 2 Transcription Factor
α‐KG: Alpha‐ketoglutarate
γH2AX: Phosphorylated H2A Histone Family Member X

## 1 Introduction

**FIGURE 1:**
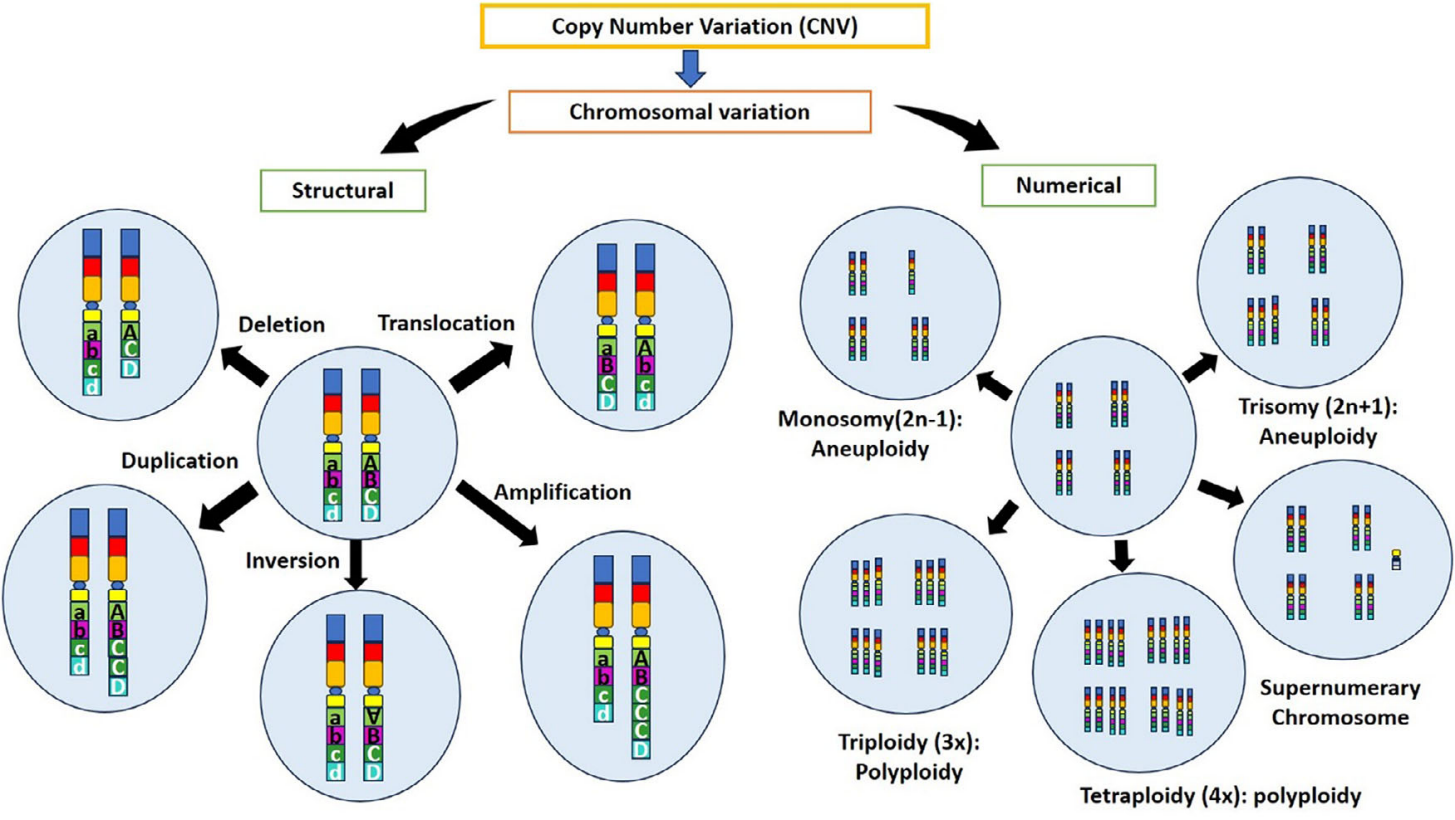
Schematic representation of chromosomal variations: Chromosomal variations can be divided into two major groups: structural and numerical. Structural variations include deletion, duplication, inversion, translocation, and amplification, which alter the physical structure of chromosomes and affect gene expression patterns. Numerical variations involve changes in chromosome number, such as aneuploidy—loss (monosomy [2n–1]) or gain (trisomy [2n+1]) of individual chromosomes—and polyploidy, which involves whole‐genome duplication (triploidy [3x], tetraploidy [4x]). The presence of a supernumerary chromosome represents an additional small chromosome beyond the normal complement. These chromosomal changes collectively contribute to genomic instability, an important hallmark of cancer and developmental disorders.

Recent advances have shown that chromosomal aberrations, either numerical or structural Figure 1 are not just random occurrences but can serve as significant indicators for cancer prognosis and therapeutic responses. Numerical aberration influences CIN, leading to frequent gains or losses of entire or large chromosomal regions over successive cell generations, Chromosomal Arm Aneuploidy (CAA) and polyploidy; affect gene dosage and expression [1]. Importantly, CIN and aneuploidy are closely related yet mechanistically distinct processes [2]. Aneuploidy refers to an abnormal chromosome number that can arise from a single chromosome mis‐segregation event, resulting in a stable but imbalanced karyotype within a clonal population. In contrast, CIN represents a dynamic process of ongoing chromosome mis‐segregation, continuously generating karyotypic diversity among tumor cells. As highlighted, CIN and aneuploidy are not synonymous but interdependent—CIN acts as a driving force that produces aneuploid cells, while the resultant aneuploidy can further exacerbate chromosomal instability by disrupting mitotic fidelity and cellular homeostasis. This reciprocal relationship sustains genetic diversity and underlies tumor evolution and adaptability [3]. Consequently, CIN‐induced heterogeneity provides a selective advantage, enabling tumor cells to adapt, metastasize, and develop resistance to therapies [4]. Numerical CIN events in glioblastoma produce a number of sub‐clones characterized by gain or loss of chromosome 7 or 10 and varying degrees of amplification of Epidermal Growth Factor Receptors (EGFR) amplification [5]. While focal amplification of EGFR gene triggers Phosphatidylinositol 3‐Kinase/Protein Kinase B (PI3K/AKT) pathway, complete gain of chromosome 7 activates Hepatocyte Growth Factor/ MET Proto‐Oncogene, Receptor Tyrosine Kinase (HGF/MET) in parallel to EGFR/PI3K signaling axis. Loss of Chromosome 10 or deletion in Chromosome 14 impairs the function of the EGFR inhibitor. Similarly, amplification of 12q13‐15 and 4q12 disrupts the function of Retinoblastoma Protein (RB) and p53 protein, and triggers various signaling pathways linked with tyrosine kinase receptors, including Kinase Insert Domain Receptor (KDR) and Platelet‐Derived Growth Factor Receptor (PDGFR). Frequent loss of function in various tumor suppressor genes, including Cyclin Dependent Kinase Inhibitor 2A / 2B (CDKN2A/2B), and Protein Tyrosine Phosphatase Receptor Type D (PTPRD), expressing cell cycle regulatory proteins associates with loss of chromosome 9P in glioblastoma [6]. In addition to numerical CIN, Double‐stranded DNA breaks with possible rearrangement increase the rate of intra‐chromosomal aberrations in structural CIN, leading to chromosome segment gains or losses, chromosomal fusion, and chromothripsis [7, 8], a catastrophic process where the lagging chromosome undergoes massive rearrangement [9]. Whole genome sequencing analysis has linked chromothripsis to the amplification of key oncogenes such as EGFR, Mouse Double Minute 2 / 4 (MDM2/4), and Cyclin‐Dependent Kinase 4 (CDK4), which promote tumorigenesis by enhancing cell proliferation, inhibiting tumor suppressor pathways, and enabling uncontrolled cell cycle progression [10, 11]. RNA sequencing studies of Isocitrate Dehydrogenase (IDH)‐wild‐type Glioblastoma Multiforme (GBM) further identified chromothripsis‐driven novel gene fusions, including the fusion of Septin‐14 (SEPT14) and Vesicular overexpressed in cancer, prosurvival protein 1 (VOPP1) with EGFR forms EGFR‐SEPT14 and EGFR‐VOPP1, respectively, which are implicated in aberrant signaling and tumor progression. These findings underscore the dual impact of chromothripsis in amplifying oncogenes and creating fusion proteins with potential oncogenic properties, highlighting its central role in the aggressive biology of GBM [12]. Thus, the resulting intra‐tumor heterogeneity driven by chromosomal alteration features the sub‐clones with different growth rates, malignant potential, resistance to therapy, propensity to invade and metastasize, and other phenotypes [5]. Such evidences attract the attention to focus on discussing the impact of CIN‐based chromosomal aberration on GBM progression, as CIN could serve as a platform to develop novel therapeutics for difficult‐to‐treat cancers [4].

## 2 Chromosomal Alteration Influences Molecular Classification of Glioblastoma and Relevant Prognosis

**FIGURE 2:**
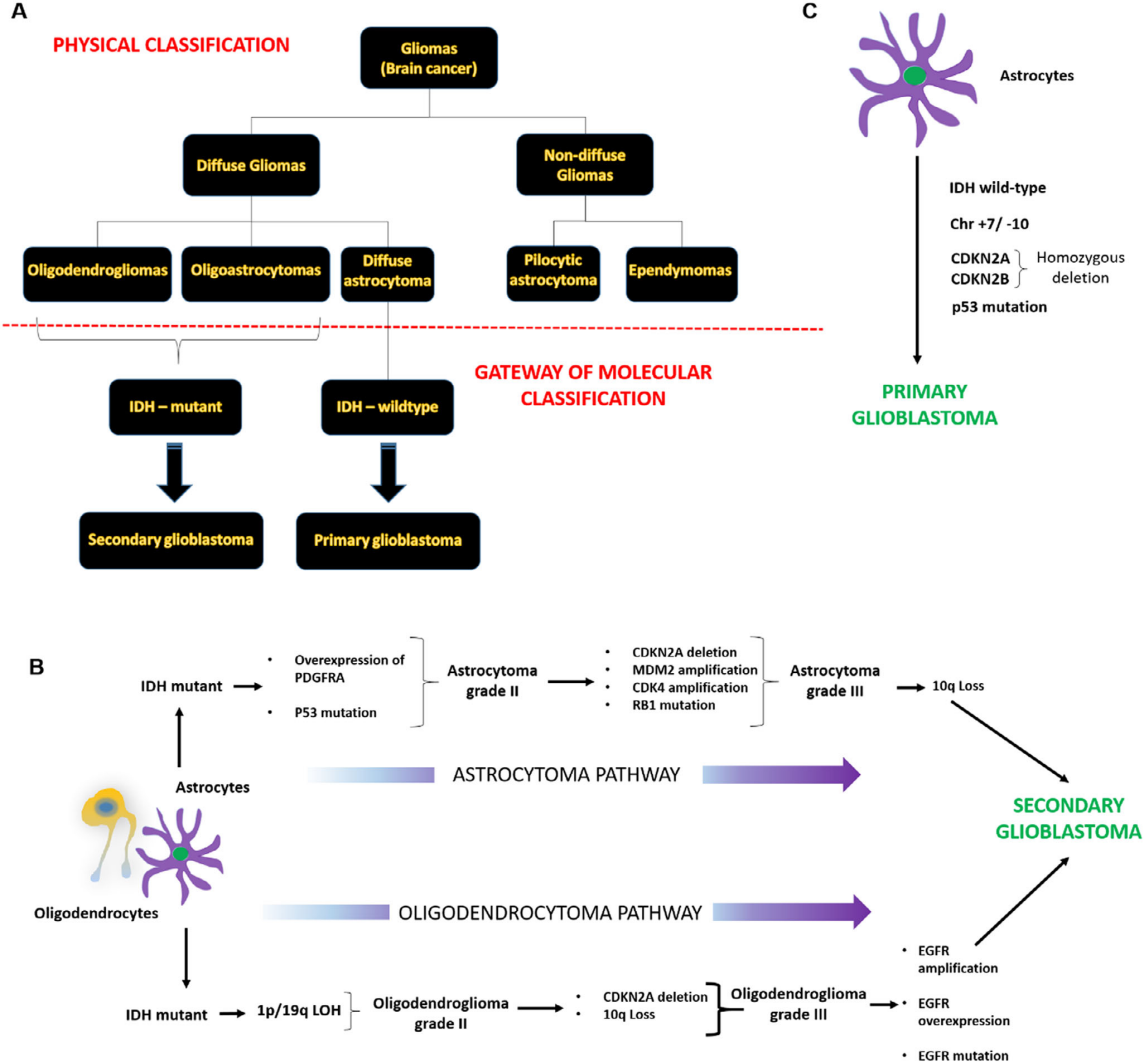
Classification of glioma and glioblastoma development pathways. A. Gliomas are physically classified based on the type of cells they originate from and their ability to invade neighboring brain parenchyma. IDH is the first line molecular marker helps in differentiating primary and secondary glioblastoma emerged from diffuse gliomas. B. and C. Demonstrating Primary and secondary glioblastoma development pathways involve mutations in cell cycle regulatory proteins (p53, and RB1), alteration in the expression of growth factor receptors, CDKN2A/2B deletion and chromosomal aberration in Chromosome 7 and 10.

Glioblastoma, alternatively known as GBM, is the most common and lethal primary malignancy of the central nervous system and is categorized into either IDH wild type or IDH mutant depending upon the mutational status in the metabolic enzyme, IDH as per 2016 World Health Organization (WHO) [13]. GBM affect primarily the cerebral hemispheres of adult brains, and specifically the brainstem region in children [14]. The Central Brain Tumor Registry of the United States (CBTRUS) statistical report demonstrates, in the U.S., the incidence rate of GBM is 3.22 per 100 000 with a median age of 64 years and account for 38.7% of Central Nervous System (CNS) tumors, with 23 000 GBM cases in India reported in 2016 [15]. It is reported males having higher incidence rate of GBM compare to females and further, increasing number of GBM cases in following years indicating a concerning upward trend in GBM incidence globally characterizes with poor survivality, usually less than 18 months in treated patients, and 5‐year survival under 5% in patients [16, 17, 18]. Recent comprehensive analyses further highlight that glioblastoma characterized by extensive intratumoral heterogeneity, genomic instability, and diffuse infiltrative growth that collectively contribute to poor clinical outcomes and limited therapeutic success [19]. Hence, for accurate identification of cancer subtypes has become a major priority for improving treatment strategies. According to the revised 2021 World Health Organization classification of tumors of the central nervous system (WHO CNS5), glioblastoma is categorized under adult‐type diffuse gliomas, which are characterized by their infiltrative growth into the surrounding CNS parenchyma, distinguishing them from circumscribed (non‐diffuse) gliomas [20]. Under the new schema, only IDH‐wildtype diffuse astrocytic tumors in adults are considered glioblastoma. Tumors that are IDH‐mutant, even with high‐grade histologic features, are now classified as astrocytoma, IDH‐mutant, WHO grade 4 rather than “glioblastoma, IDH‐mutant” (WHO CNS5) [20, 21]. Additionally, molecular criteria have gained importance: an IDH‐wildtype diffuse astrocytoma lacking classic histologic grade IV features (necrosis, microvascular proliferation) may nevertheless be classified as glioblastoma if one or more of the following are present: Telomerase Reverse Transcriptase (TERT) promoter mutation, EGFR amplification, or combined +7/−10 chromosomal copy number alterations [21, 22] demonstrated in Figure 2. Therefore, the ‘AMEN’ score–based histological characterization including nuclear Atypia, Mitosis index, Endothelial cell proliferation, and Necrosis proposed within the WHO glioblastoma classification framework may be insufficient to clearly distinguish between primary and secondary GBM. In addition, inter‐observer variability among pathologists can lead to inconsistent diagnoses [23]. Hence integrating the knowledge of histology with molecular profiles including IDH mutant variance and chromosomal aberrations of CNS tumors, facilitates recognition of distinct molecular subtypes, tumor heterogenity and spatial variability [20]. Therefore, introduction of the 2021 WHO classification system compensates the limitation of 2016 WHO classification, which was based on histopathological grading, IDH mutation and 1p/19q codeletion status as key molecular markers failing clearly distinguish GBM subtypes [24, 25]. Thus, in parallel of considering mutational status in IDH and histological features, chromosomal alteration supports diagnostic precision of GBM in patients.

## 3 Chromosomal Alteration in Astrocytoma, IDH‐Mutant, WHO Grade 4

Astrocytoma, IDH‐mutant, CNS WHO grade 4 comprises the subset of IDH‐mutant diffuse astrocytic neoplasms that either display classic grade‐4 histology (necrosis and/or microvascular proliferation) or harbor molecular high‐risk alterations—most notably homozygous deletion of CDKN2A/B, which alone is sufficient to designate an IDH‐mutant tumor as WHO grade 4 under the 2021 WHO criteria [20, 26]. Clinically and molecularly, this category sits at the upper end of the IDH‐mutant astrocytoma spectrum (grades 2–4) and typically includes tumors with the canonical co‐occurrence of IDH1/2 mutation, Tumor Protein 53 (TP53) mutation, and Alpha‐Thalassemia/Mental Retardation Syndrome X‐linked (ATRX) loss. Within grade 4 IDH‐mutant astrocytomas, one also observes subgroups defined by additional focal copy‐number alterations such as CDKN2A/B homozygous deletion, CDK4/Cyclin D2 (CCND2) amplification, MDM2/MDM4 alterations, and focal gains or losses that are acquired during malignant progression [27, 28, 29]. Cytogenetically, these tumors generally demonstrate a lower frequency of the classical +7/−10 whole‐chromosome signature and EGFR amplification that typify IDH‐wildtype glioblastoma, but they accumulate focal Copy Number Variations (CNVs) and region‐level aneuploidy (including chromosome 9p loss) as they progress to grade 4, producing an elevated copy‐number burden and subclonal chromosomal instability in many cases [28, 30]. Functionally, these chromosomal events translate into predictable protein‐level consequences, including CDKN2A/B loss, which causes loss of p16^INK4a^ and p14^ARF^ protein expression with resultant unchecked CDK4/6–RB pathway activity and impaired p53‐mediated checkpoints. Second, ATRX loss associates with perturbation of chromatin remodeling and Alternative Lengthening of Telomeres (ALT) phenotypes; MDM2/MDM4 alterations blunt p53 signaling, and CDK4/CCND2 amplifications increase cyclin‐dependent kinase activity — all changes readily detectable by immunohistochemistry or proteomic profiling and concordant with the underlying CNV landscape [26, 27]. Mechanistically, mutant IDH enzymes (typically IDH1 R132H) produce the oncometabolite D‐2‐hydroxyglutarate (2HG). This metabolite competitively inhibits α‐ketoglutarate–dependent dioxygenases, including Ten‐Eleven Translocation (TET) DNA demethylases and histone lysine demethylases, resulting in widespread epigenetic reprogramming and the establishment of a glioma‐CpG island methylator phenotype (G‐CIMP). Such epigenetic remodeling has been shown to alter chromatin architecture and increase heterochromatin‐associated replication stress, which slows replication fork progression and promotes DNA damage accumulation during tumor evolution. Recent mechanistic studies further indicate that oncogenic IDH mutations promote heterochromatin‐mediated replication stress without completely abolishing homologous recombination (HR) repair, thereby creating genomic conditions favorable for the gradual accumulation of copy‐number alterations and chromosomal instability in glioma cells [31]. Consequently, persistent replication stress and impaired DNA damage responses facilitate the accumulation of double‐strand DNA breaks and suboptimal repair processes, ultimately promoting chromosomal instability (CIN) and aneuploidy during astrocytoma progression. However, 2HG‐mediated homologous recombination (HR) defects and replication stress not only contribute to the emergence of copy number variations (CNVs), such as CDKN2A/B loss, during malignant progression, but also create exploitable therapeutic vulnerabilities. These include increased sensitivity to poly(ADP‐ribose) polymerase (PARP) inhibitors, as well as strategies targeting replication stress and cell‐cycle checkpoints. Additionally, these alterations provide a rationale for CDK4/6 inhibition in tumors harboring CDKN2A/B loss or CDK4 amplification, which are currently under active preclinical and clinical investigation [32, 33]. These changes, influenced in part by IDH‐mutation‐mediated epigenetic and replication perturbations [13, 34], drive malignant progression across the diffuse glioma spectrum and support the development of highly aggressive glioma states, including glioblastoma [6, 35], as illustrated in Figure 2A,B. Consistent with recent integrative genomic analyses of glioma evolution, the accumulation of focal CNVs, chromosomal alterations, and epigenetically driven genomic instability represents a key molecular mechanism underlying the progression of lower‐grade IDH‐mutant astrocytomas toward higher‐grade disease states [34]. Taken together, the genomic architecture of astrocytoma, IDH‐mutant, WHO grade 4 reflects progressive chromosomal instability and accumulation of focal copy‐number alterations during tumor evolution. Hence, documenting these genomic and proteomic changes is critical for accurate WHO grading, prognosis, and for selecting targeted therapeutics.

## 4 Chromosomal Alteration in IDH‐Wildtype Diffuse Astrocytic Tumors (Glioblastoma)

Although IDH‐mutant astrocytoma harbors a broad range of genomic instability, distinct metabolic weaknesses stemming from disrupted Tricarboxylic Cycle Acid (TCA) cycle activity, reduced proliferation rates, and modified cellular metabolism [36]. As a result, IDH –mutant associates with better prognosis compared to its counterpart, IDH –wildtype, and considered as a decisive marker for secondary GBMs [37]. Unlike secondary glioblastomas, Primary glioblastomas, which are largely IDH‐wildtype, are cytogenetically distinguished by recurrent large‐scale chromosomal abnormalities, most notably gain of chromosome 7 and loss of chromosome 10 (+7/−10), a characteristic aneuploidy signature observed in the majority of tumors [38] (Figure 2C). Alternatively termed, IDH‐wildtype diffuse astrocytic tumors constitute the most aggressive category of adult‐type diffuse gliomas. According to the 2021 WHO Classification of Tumors of the Central Nervous System (CNS5), these tumors are classified as glioblastoma, IDH‐wildtype, CNS WHO grade 4, which exhibit defined molecular criteria, including TERT promoter mutation, EGFR amplification, or the combined whole‐chromosome +7/−10 signature even in the absence of grade‐4 histological characteristics [20]. Molecularly, these tumors display extensive chromosomal instability (CIN) and widespread aneuploidy accompanied by focal genomic alterations affecting major oncogenic signaling pathways. Recurrent events include EGFR amplification (7p11.2), MDM2 and CDK4 co‐amplification (12q13‐15), Platelet‐Derived Growth Factor Receptor Alpha (PDGFRA) amplification (4q12), Phosphatase and Tensin Homolog (PTEN) loss or mutation (10q23.31), CDKN2A/B homozygous deletion (9p21.3), and RB1 deletion (13q14), which collectively reshape oncogenic signaling networks and drive tumor progression [5, 22, 39].

These large‐scale chromosomal aberrations drive oncogenic signaling, disrupt cell‐cycle checkpoints, and alter the proteomic landscape of tumor cells. For instance, EGFR amplification results in overexpression and constitutive activation of EGFR protein and downstream PI3K–AKT–mammalian target of Rapamycin (mTOR) and RAS/Rapidly Accelerated Fibrosarcoma/Mitogen‐Activated Protein Kinase (RAS–RAF–MAPK) cascades, associated with cellular proliferation, metabolic adaptation, and survival signaling [40]. Concurrently, PTEN loss eliminates a critical negative regulator of PI3K signaling, further amplifying oncogenic pathway activity and contributing to tumor growth and therapy resistance [41]. Similarly, CDKN2A/B deletion leads to reduced p16^INK4a^ and p14^ARF^ expression, thereby releasing inhibition on CDK4/6–RB and p53 pathways, respectively, further enabling cell‐cycle progression and genomic instability [34]. In addition to focal alterations, the characteristic +7/−10 chromosomal signature alters gene dosage across numerous genomic loci and has been associated with increased mitotic activity, intratumoral heterogeneity, and resistance to therapy [38]. Collectively, these chromosomal and proteomic alterations define the aggressive biology of IDH‐wildtype astrocytic tumors, linking CIN‐driven aneuploidy to poor prognosis and underscoring the need for therapies targeting mitotic fidelity, replication stress, and EGFR/PI3K signaling dependencies [19, 42]. Integration of chromosomal alterations with molecular biomarkers and epigenetic features (Table 1) therefore, improves diagnostic precision and prognostic stratification in patients with IDH‐wildtype glioblastoma [43].

**TABLE 1:** Molecular prognostic markers in glioblastoma.

Molecular markers	Functions	Prognosis
IDH mutation [19, 20]	Convert alpha‐ketoglutarate to D‐2‐hydroxyglutarate.	Common in younger patients and secondary GBM. IDH‐mutant tumors have a better prognosis than IDH‐wild type GBM.
Methylation of MGMT promoter [166]	A DNA repair protein called MGMT eliminates alkyl groups from guanine's O6 position in DNA, promoting cells' resistance to the alkylating chemical TMZ.	Methylation at promoter region results in MGMT silencing, which obstructs DNA repair and predicts better response to TMZ and improved survival.
TERT promoter mutations [167]	TERT involves in telomere maintenance.	Mutation in TERT exerts lead to increased telomerase activity, promoting tumor cell immortality. TERT promoter mutation/IDH‐wildtype but a substantial association with EGFR amplification contribute to worse survival outcome
EGFR amplification [43]	It is a transmembrane tyrosine kinase receptor and associate with cell growth, motility and survival.	EGFR amplification underscores poorer overall survival in IDH‐diffuse gliomas and GBM EGFR stands as an independent prognostic marker for GBM
ATRX [5, 19]	ATRX is involved in telomere stability and length regulation, preventing telomere dysfunction.	In GBMs, ATRX mutations are linked to an alternate telomere lengthening phenotype. ATRX mutations, when combined with IDH mutations, often result in a less aggressive tumor phenotype with longer survival.

IDH: Isocitrate dehydrogenase, MGMT: O^6^‐methylguanine‐DNA methyltransferase, TERT: Telomerase Reverse Transcriptase, EGFR: Epidermal Growth Factor Receptor, ATRX: Alpha‐Thalassemia/mental Retardation Syndrome X‐linked.

## 5 Mis‐Segregation – Driven CIN in Glioblastoma

CIN‐driven chromosomal abnormality plays a crucial role in gliomas, adding to their complexity, course, and treatment difficulties. CIN‐driven disruption of genetic content distribution benefits cancer progression by enabling neoplastic cells to bypass regulatory checkpoints. Replication errors, environmental toxins, and reactive oxygen species challenge genomic integrity, and while cells possess DNA repair mechanisms, persistent CIN breaches these defenses, allowing sub‐clonal diversity that fosters therapy resistance [4, 44]. Improper attachment of chromosomes to spindle microtubules and subsequent defects in the spindle assembly checkpoint (SAC) promote chromosome missegregation during mitosis, thereby driving CIN across successive cell divisions [45].

## 6 Merotelic Attachment Drives Chromosomal Mis‐Segregation

**FIGURE 3:**
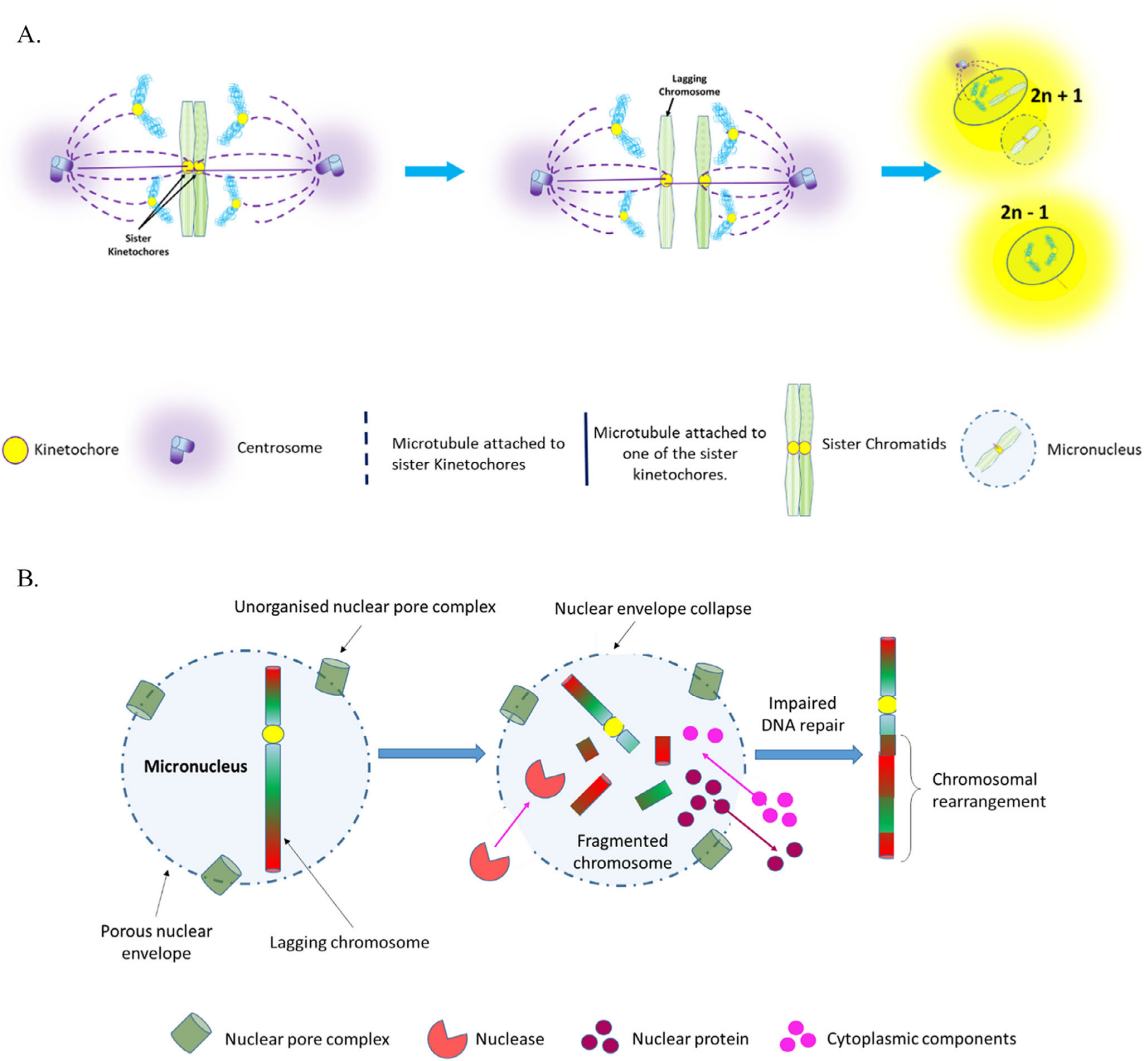
Illustrating Merotelic attachment – driven CIN. A. Demonstrating Merotely – driven CIN in diploid cells (2n). Merotelic attachment gives rise the lagging chromosome that results one of the diploid cells gaining one whole chromosome (2n + 1) and a micronucleus due to the mis‐segregation of sister chromatids. B. Representing the steps of chromothripsis in micronucleus. The structure of nuclear envelope surrounding micronucleus is characterized as unorganized nuclear pore complex, porous and tend to rapture. The compromised structure of nuclear envelope allows exchanging of nuclear and cytoplasmic components that consequently expose DNA to the cytoplasmic entities including nucleases results in chromosomal fragmentation. Impair DNA damage response forcibly stitches fragmented segments randomly leading to chromosomal rearrangement, that is, chromothripsis.

Merotelic attachment is constituted of linking one sister kinetochore to both spindle poles, which leads to uneven distribution of chromosomes in successive daughter cells [46], Figure 3A. Merotelic attachment is a subtle yet pervasive defect in kinetochore–microtubule (K–MT) interactions that represents a major driver of CIN in cancer. Under normal mitosis, each sister kinetochore attaches to microtubules from opposite spindle poles (amphitelic attachment), ensuring accurate chromosome segregation. In contrast, during merotelic attachment, a single kinetochore aberrantly binds microtubules from both poles, creating improper tension across the centromere [46, 47]. Because kinetochores in merotelic attachments remain bound to spindle microtubules and can generate apparent tension, these erroneous attachments often evade surveillance by the spindle assembly checkpoint (SAC). Consequently, cells may prematurely enter anaphase with unresolved kinetochore–microtubule misattachment, increasing the likelihood of lagging chromosomes and chromosomal instability [46, 48].

**FIGURE 4:**
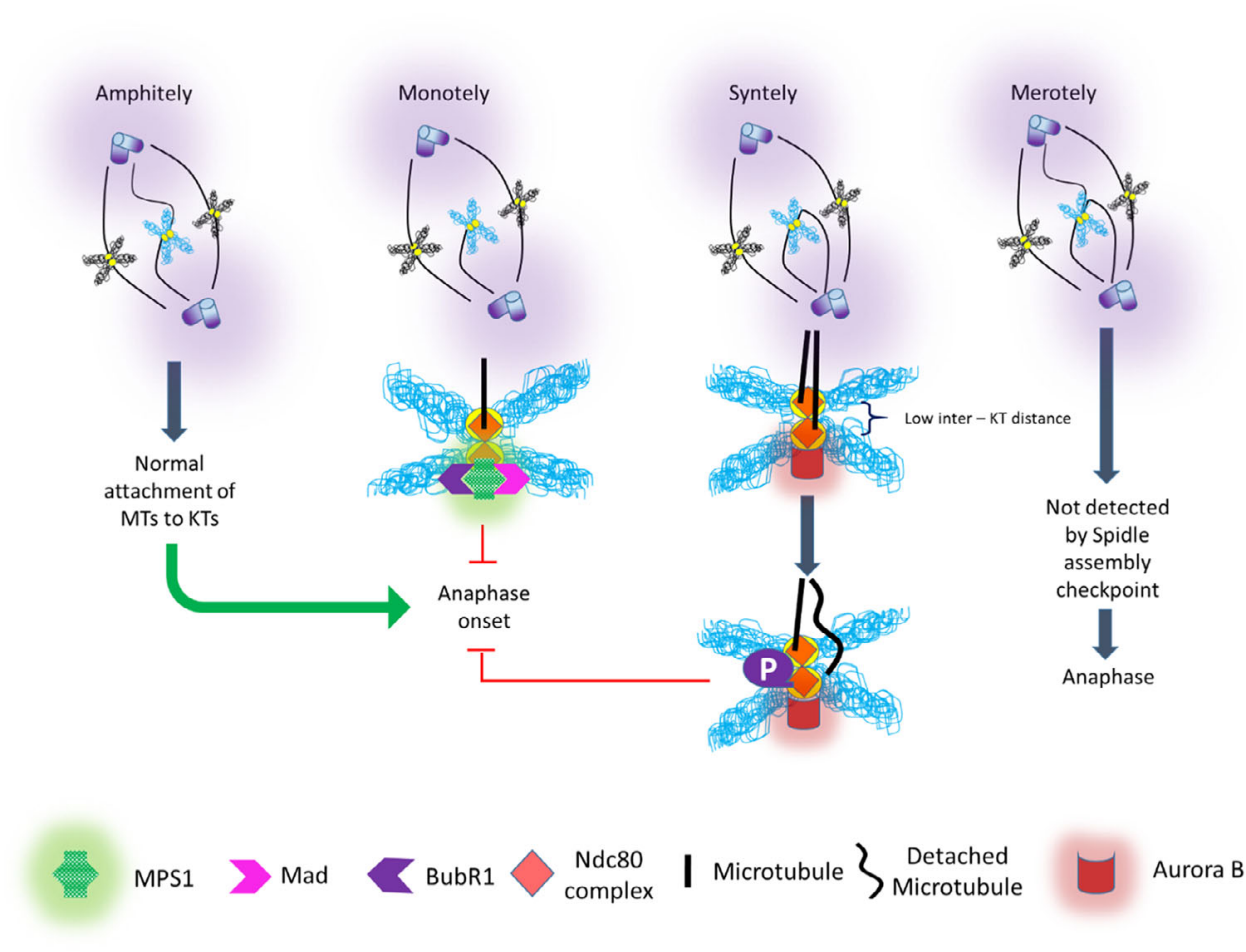
Representing different types of KT‐MT attachment and SAC proteins associated with surveillance mechanism. Ndc80 complex at KT serves as an attachment site of MT emerged from centrosomes. Unlike Amphitelic connection, Monotely, Syntely and Merotely demonstrating erroneous attachment between KT and MT. Monotely and Syntely connection prohibit the onset of anaphase through signaling molecules including MPS1, Mad, BubR1 and Aurora B. However, Merotely connection remains unchecked by SAC system and the error is encouraged in anaphase. Chromosome is highlighted in blue.

As illustrated in Figure 4, SAC acts as a surveillance mechanism, that prevents anaphase onset until all kinetochores are properly attached to spindle microtubules. Central to this process is the Nuclear Division Cycle 80 (Ndc80) complex, a key outer‐kinetochore microtubule‐binding complex that integrates mechanical attachment and checkpoint signaling to ensure faithful chromosome segregation [49]. The SAC cascade begins with the recruitment of Monopolar Spindle 1 Kinase (Mps1) kinase to kinetochores lacking improper microtubule attachment observed in Monotely attachment type, where one sister kinetochore remains unattached by spindle fibre [50]. Mps1 sequentially phosphorylates kinetochore proteins such as Kinetochore Scaffold 1 (Knl1), Budding Uninhibited by Benzimidazoles 1 (Bub1), and Mitotic Arrest Deficient 1 (Mad1). This phosphorylation cascade facilitates the recruitment and assembly of checkpoint complexes, including Bub1–Bub3 and Budding Uninhibited by Benzimidazoles‐Related 1‐ Budding Uninhibited by Benzimidazoles 3 (BubR1–Bub3), at unattached kinetochores [51, 52]. Bub1 serves to recruit the Mad1–Mad2 complex, which amplifies SAC signaling, while BubR1 plays a critical role in stabilizing the Mitotic Checkpoint Complex (MCC) and inhibiting the Anaphase‐Promoting Complex/Cyclosome (APC/C) through Cell Division Cycle 20 homologue (Cdc20) binding [53]. Further studies explores the relationship between inter‐kinetochore (KT) stretching and the SAC. Inter‐kinetochore stretching contributes to the discrimination between proper bi‐oriented attachments (amphitelic) and erroneous syntelic attachments; low tension at syntelic kinetochores allows Aurora B kinase to phosphorylate outer kinetochore substrates such as the Ndc80 and Mis12 complexes, destabilizing kinetochore–microtubule interactions and promoting error correction [54]. However, incomplete occupancy of Ndc80 complex by MTs promotes merotelic attachment, which is undetectable by the SAC signaling cascade due to the following inter‐KT distance exerted by the spindle fibre on one of the properly attached sister kinetochores. Thus, maximising the risk of lagging chromosomes, a major cause of CIN [55]. Recent studies show that hyperstabilized K–MT interactions in tumor cells hinder turnover necessary for correcting merotely, reinforcing persistent missegregation [56, 57]. Moreover, large‐scale genomic analyses indicate that recurrent whole‐chromosome aneuploidies in cancer frequently originate from merotelic nondisjunction events [58]. Thus, merotelic attachment constitutes a checkpoint‐blind mechanism linking spindle dysregulation to CIN‐driven tumor evolution and intratumoral heterogeneity.

Although the contribution of proteins regulating merotelic kinetochore–microtubule attachment to glioblastoma progression remains incompletely characterized, chromosome segregation errors frequently generate lagging chromosomes that fail to be incorporated into the daughter nuclei and instead form small extranuclear structures known as micronuclei (MN). Chromosomes trapped within micronuclei are highly prone to DNA damage and fragmentation, which can subsequently lead to catastrophic genomic rearrangements such as chromothripsis, thereby contributing to tumorigenesis and cancer genome evolution [59]. Figure 3B. Cell culture –based studies demonstrate exhaustion of lamin protein, specifically Lamin‐B1 (LmnB1) contributes to nuclear envelope collapse in MN [60] despite the acquisition of nuclear envelope repairing protein, Endosomal Sorting Complexes Required for Transport‐III (ESCRT –III) [61]. Further, experimental approach reveals ESCRT‐ III undergoes autophagic degradation driven by autophagic receptor p62/Sequestosome1 (SQSTM1) [62] that ultimately exposes DNA to the cytoplasmic components result in DNA damage and chromosome fragmentation [61, 63]. Further, sub‐optimal function of DNA repair machineries in MN promotes chaotic chromosomal rearrangement or chromothripsis [64]. Chromothripsis is more frequent in glioblastoma, with extensive rearrangements of one or more chromosomes and, therefore, CNV of genes occurs [11] resulting focal amplification of oncogenic driver genes frequently arising from complex genomic rearrangements that generate high‐copy oncogenic amplicons and extrachromosomal DNA, thereby promoting tumor progression and genomic heterogeneity [65]. Pan‐cancer analyses have demonstrated that chromothripsis is unevenly distributed across chromosomes and cancer types. Large‐scale whole‐genome sequencing studies identified recurrent chromothriptic involvement of specific chromosomes and a high prevalence of chromothripsis in tumors such as glioblastoma [66]. This signifies chromothripsis is just not a random process but may be chromosome specific event. Kinetochore size may play a decisive factor for a chromosome to be associated with merotelic‐attachment. Indian muntjac fibroblast‐based studies demonstrate chromosomes with larger kinetochores are more susceptible to incorrect merotelic kinetochore–microtubule attachments [67]. Although the relevant study is not performed in human cells, but a single kinetochore has the capacity to bind with 12 – 24 microtubules for reliable bi‐oriented spindle attachment in the presence of functional proteins responsible for Kinetochore‐spindle fibre attachment in human cells [68]. Hence, in mechanical context it is not surprising, larger size of kinetochore likely encourage erroneous kinetochore‐spindle attachment in mitotic cycle.

Upon detecting cytosolic DNA from micronuclei rupture, cGAS synthesizes cyclic guanosine monophosphate–adenosine monophosphate (cGAMP), which activates STING, leading to the production of type I interferons and pro‐inflammatory cytokines [69, 70]. This activation fosters an anti‐tumor immune response by enhancing dendritic cell activation, promoting T cell priming, and augmenting the efficacy of immune checkpoint inhibitors [71]. However, in GBM, epigenetic modifications such as hypermethylation of STING suppresses its activity, contributing to immune evasion [72]. The activity of cGAS/STING is influenced by PTEN expression level as well. PTEN mutations in GBM compromise the STING pathway results in reduction of type I interferons levels impairing the immune‐stimulating system. Hence, it appears activating the cGAS‐STING pathway could potentially convert the “cold” immune microenvironment of glioblastoma into a “hot” tumor, enhancing immunotherapy efficacy [73]. In contrast, chronic activation of cGAS/STING can create an immunosuppressive microenvironment [74] by recruiting Myeloid‐Derived Suppressor Cells (MDSCs) and tumor‐associated macrophages, dampening effective anti‐tumor immunity [75]. Thus, mis‐segregation‐driven chromothripsis and aberrant spindle checkpoint regulation not only fuel genetic chaos but also reshape the tumor microenvironment through cGAS–STING signaling perturbations, supporting cancer cells to evade immune attack. Understanding this intricate interplay unveils CIN not merely as a byproduct of malignancy but as a therapeutic vulnerability—one that can be exploited through strategies that restore mitotic fidelity, modulate DNA repair responses, and reawaken suppressed innate immune pathways. Future therapies targeting CIN‐driven glioblastoma must thus integrate precision genome surveillance with immunomodulatory approaches, transforming chromosomal chaos from a hallmark of tumor resilience into a targetable regime.

## 7 Supernumerary Centrosomes – Driven CIN in Cancer

**FIGURE 5:**
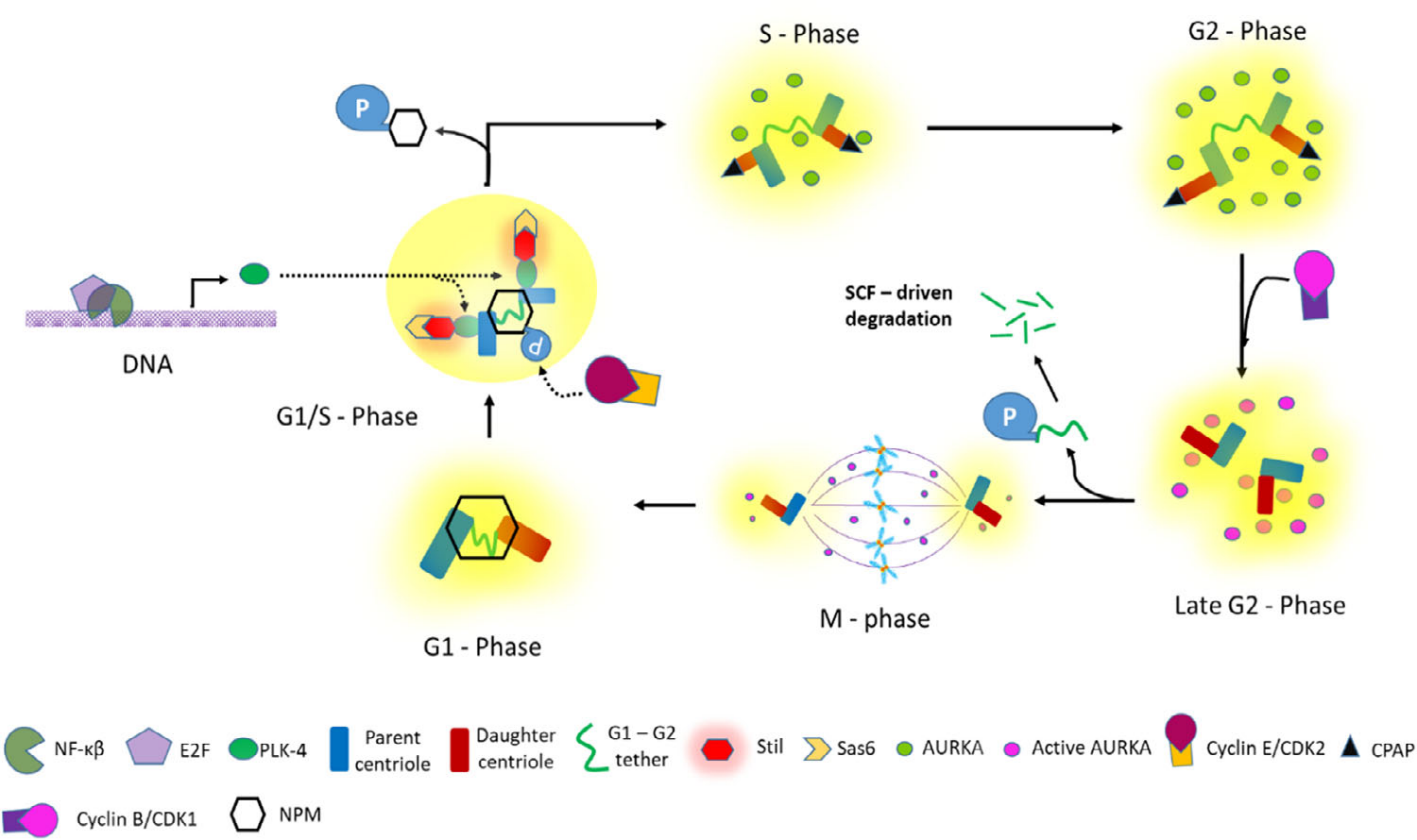
Centrosome duplication cycle in human cells. In S – phase, Cdk2/cyclin E phosphorylates key protein NPM [77] initiating the formation of daughter centrioles (brown rod) perpendicularly from parental centrioles (blue rods) aided by sequential recruitment of PLK4, Still and Sas6. Cdk2/cyclin A (not shown) and Centrosomal P4.1 associated protein (CPAP) facilitates elongation of daughter centriole in S and G2 –phase [90]. In late G2 –phase, Cdk1/cyclin B phosphorylates the G1‐G2 tether and programmed for Skp, Cullin, F‐box containing complex (SCF)‐driven proteosomal degradation allowing centriole disengagement followed by moving Parent‐daughter centriole (Brown –blue rod) pairs to the opposite side of the cells [77, 81]. This polarization of centriole pair is regulated by AURKA, thus, supporting the formation of mitotic spindle fibers require for chromosomal arrangement in M –phase. Finally, at post‐mitotic phase each daughter cell gains a Parent‐daughter centriole connecting with NPM surrounded by pericentriolar matrix (yellow) and wait for the next round of duplication cycle. Transcription factors including NF‐κβ and E2 Promoter Binding Factor (E2F) acts as transcriptional activators for PLK‐4 [90].

Further, supernumerary centrosomes are additional causes play roles in promoting CIN in cancer cells. Cancer cells frequently possess supernumerary centrosomes and depend on centrosome clustering to ensure proper division, a process that contributes to CIN by promoting the formation of merotelic kinetochore attachments. CIN drives alterations in the sequence and copy number of oncogenes, enabling cancer cells to adapt and evolve [76]. As illustrated in Figure 5, in normal cells, during S‐phase of cell cycle, Cyclin E/CDK2 initiates centrosome duplication by phosphorylating Nucleophosmin (NPM), detaching it from the centrosome as the non‐phosphorylated form of NPM inhibits premature splitting of centriole [77]. Next, Cyclin B/CDK1 supports later stages of centrosome maturity by phosphorylating proteins including kinesin‐5 family motor protein (Eg5) and NIMA Related Kinase 2 (Nek2), dissolving centriole linkers (G1‐G2 tether) constitute of C‐Nap1 and rootletin [78, 79] for proper mitotic spindle assembly [80]. This agrees with the observations where consistent loss of NPM or structural regulator of NPM, that is, Inhibitor of NF‐kβ kinase α (IKKα) linked with centrosome amplification results in the disruption of genome integrity contributing tumor aggression [81]. In addition, overexpression of pro‐centriolar biogenesis factors, Polo‐Like Kinase 4 (PLK4), Human Spindle Assembly abnormal protein 6 homolog (HsSAS‐6), and Stromal Tumor Infiltrating Lymphocytes (STIL) directly related with centrosome amplification [82, 83] as reported in different cancer types, including GBM [80, 84].

**FIGURE 6:**
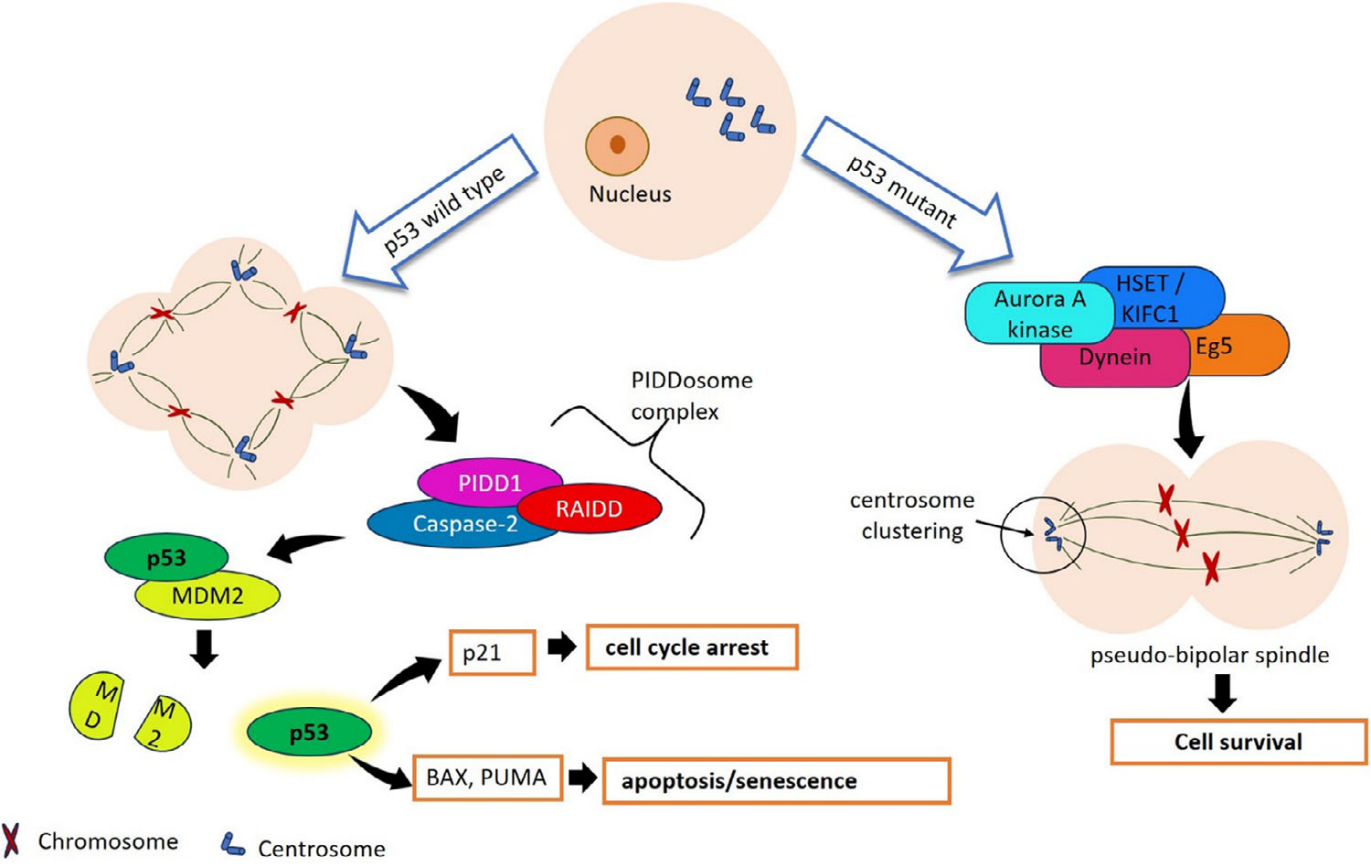
Differential cellular responses to centrosome amplification in p53 wild‐type and its mutant counterpart. The schematic illustrates how p53 status determines the fate of cells harboring supernumerary centrosomes. In cells with wild‐type p53, centrosome amplification is sensed through the PIDDosome–caspase‐2–MDM2–p53 signaling axis, leading to stabilization and nuclear accumulation of p53. Activated p53 either induces cell cycle arrest (via p21) or apoptosis (via BAX, PUMA), thereby preventing proliferation of aneuploid cells and maintaining genomic integrity. In contrast, in p53‐mutant or p53‐deficient cells, this checkpoint fails to activate, allowing continued cell division despite centrosome amplification. These cells evade mitotic catastrophe by engaging centrosome clustering mechanisms involving Aurora A kinase, HSET/KIFC1, Eg5, and dynein, which bundle multiple centrosomes into two spindle poles to form a pseudo‐bipolar spindle. This results in cell survival and permits chromosome segregation errors resulting in CIN.

Centrosome amplification (CA) is a recurrent cytological hallmark of GBM that converges on a discrete set of centrosome‐associated regulators whose dysregulation promotes invasion through both cell‐autonomous cytoskeletal remodeling and non‐cell‐autonomous disruption of tissue architecture. Core centriole/centrosome factors such as PLK4 are frequently upregulated in high‐grade gliomas and drive centriole overduplication, genomic instability, and pro‐invasive phenotypes while also promoting radioresistance in GBM models [85]. Centrosome‐localized scaffolds and abscission regulators including Centrosomal protein of 55 KDa (CEP55) are overexpressed in glioma and enhance migration, matrix‐remodeling proteases, and stem‐like neurosphere formation via PI3K/AKT– Forkhead Box M1 Signaling Axis (FOXM1) and Nuclear Factor Kappa‐light‐chain‐enhancer of Activated B cells (NF‐κB) signaling, linking CA to enhanced invasion and stemness [86]. Apart from PLK4, the association of NEK2 with GBM progression was investigated. Overexpression of NEK2 promotes the accumulation of NF‐κβ in the nucleus results in transcription of genes involved in inflammation, cell survival, proliferation, and metastasis, thereby contributing to glioblastoma progression [87]. Nevertheless, supernumerary centrosome does exist in normal cells but stabilization of p53 exert cell cycle arrest and induce apoptosis. In contrast, Loss or mutation of p53 disrupts the surveillance mechanisms that normally restrict centrosome amplification. Consequently, cancer cells harboring supernumerary centrosomes can cluster them to form pseudo‐bipolar spindles, allowing escape from cell‐cycle arrest while increasing chromosome missegregation and ultimately promoting chromosomal instability (CIN) [76, 88], as illustrated in Figure 6. Centrosomal clustering begins with Eg5‐aided maneuvering of supernumerary centrosomes apart results in jeopardizing chromosomal attachment with spindle fibers that overwhelm SAC surveillance mechanism [76]. Next, coordination of various proteins, including microtubule motor proteins like Human Kinesin Family Member C1 (HSET) (also known as KIFC1) and actin‐dependent forces to bundle excess centrosomes into two functional spindle poles [89]. Proteins such as Intraflagellar Transport (IFT) interact with HSET to stabilize these clusters [90], while Aurora Kinase A (AURKA)‐driven phosphorylated Nuclear Mitotic Apparatus (NuMA) protein anchors centrosomes at the spindle poles, ensuring bipolar spindle formation [91, 92]. Mitotic kinases that control centrosome maturation—notably AURKA—additionally couple centrosome function to actin/vimentin reorganization and Rho‐GTPases signaling, enabling chemokine‐directed migration and periventricular invasion [93]. AURKA ensures mitotic fidelity by recruiting its substrate, Transforming Acidic Coiled‐Coil Containing Protein 3 (TACC3) for stabilizing kinetochore fibers [94]. However, Overexpression of TACC3 in glioblastoma drives multiple pro‐tumorigenic programs including enhanced proliferation, migration/invasion, stem‐like properties, and survival — in part through its roles at centrosomes, spindles, and in mitotic centrosome‐clustering networks, making TACC3 both a marker of aggressive disease and a therapeutic vulnerability [95]. Interestingly, in its fusion form with Fibroblast growth factor receptor (FGFR), notably identified as FGFR3–TACC3 fusion, exerts a dual pathogenic effect in GBM. Constitutive FGFR kinase signaling that promotes growth and survival, plus structural disruption of mitotic machinery via the TACC3 moiety — sequestering endogenous TACC3 away from the spindle, results in destabilizing the microtubule dynamics, increasing chromosome misalignment/missegregation, and promoting aneuploidy and chromosomal instability [95, 96]. Mechanistic studies show that CA alone is sufficient to perturb cell–cell junctions, remodel the extracellular matrix, and engage Rap1/adhesion signaling to enable collective and single‐cell invasion, providing a unifying link between centrosome deregulation and the highly infiltrative phenotype of GBM [97]. Hence, centrosomal clustering promoting proteins could be potential therapeutic targets to selectively induce cell death in tumor cells [98].

## 8 Impaired DNA Repair Mechanism Promotes CIN

Defects in fundamental DNA‐repair pathways—such as HR, non‐homologous end joining (NHEJ), base‐excision repair (BER), and mismatch repair (MMR)—lead to replication stress and accumulation of DNA double‐strand breaks (DSBs), producing aneuploidy and CIN through mis‐segregation, micronucleus formation, and chromosome bridge breakage [34, 56]. Notably, rare gliomas harboring germline or somatic mutations in the DNA polymerase genes POLE and POLD1—key enzymes responsible for DNA replication fidelity and proofreading—exhibit profound replication errors that drive chromosomal instability and give rise to extreme hypermutated tumor phenotypes [5]. Once established, persistent aneuploidy exacerbates DNA‐repair dysfunction by inducing haploinsufficiency of key repair genes (e.g., Breast Cancer gene 1/2 (BRCA1/2), Radiation sensitive protein 51 (RAD51), Meiotic Recombination 11 (MRE11), ATRX, Ataxia‐telangiectasia mutated (ATM)) and proteotoxic stress, which destabilizes repair protein networks and impairs DNA Damage Response (DDR) signaling fidelity [99, 100]. Aneuploid cells also exhibit micronuclear entrapment of chromosomes, where aberrant replication and nuclear envelope rupture cause defective recruitment of repair proteins (e.g., Mediator of DNA damage checkpoint 1 (MDC1), 53BP1, BRCA1), leading to chromothripsis and large‐scale structural rearrangements highlighted from the accumulation of Phosphorylated H2A Histone Family Member X (γH2AX) in the MN, a marker for earliest cellular responses to the formation of DSBs [101, 102]. These self‐reinforcing cycles of repair deficiency and karyotypic chaos are particularly pronounced in GBM, where loss of ATM, ATRX, MutS homolog 6 (MSH6), or O6‐methylguanine‐DNA methyltransferase (MGMT) promoter methylation‐mediated silencing increases both mutation burden and chromosomal mis‐segregation [34]. In parallel, aneuploidy‐associated replication stress activates Ataxia Telangiectasia and Rad3‐related protein – Checkpoint kinase 1 (ATR–CHK1) and WEE1 G2 checkpoint kinase (WEE1) checkpoints as compensatory survival pathways, which have emerged as therapeutic vulnerabilities in GBM with DNA‐repair deficiencies [56, 103]. Targeting these compensatory DDR nodes—through PARP inhibitors in HR‐deficient tumors, or ATR/CHK1/WEE1 inhibitors to induce mitotic catastrophe in CIN‐positive GBM—represents a promising synthetic‐lethal approach [101, 104]. Therefore, the intersection of DNA‐repair impairment, aneuploidy, and CIN defines a key evolutionary axis in GBM, where genomic chaos both drives malignancy and exposes actionable molecular liabilities that can be therapeutically exploited.

## 9 Chromosomal Alteration Promotes Stemness and Invasion in Glioblastoma

Cancer stem cells (CSCs) drive intra‐tumor heterogeneity through their dual ability to self‐renew and differentiate into diverse tumor cell types. Their plasticity enables transitions between stem‐like and non‐stem‐like states in response to environmental cues, allowing tumors to adapt to stresses, including therapeutic interventions, and contributing to treatment resistance [105]. The detrimental effect of stemness is consistent in brain cancers, where enrichment of CSCs known as GSCs, expressing nestin, CD133, and CD163 cell surface markers [14] in the tumor niche, exerts poor prognosis in patients [106] despite targeting signaling axes involved in GBM pathogenesis [107].

CIN contributes to tumor heterogeneity [108] through generating CNVs determined by single‐cell RNA sequencing (scRNA‐seq) in a tumor [109, 110], which promotes the emergence of genetically distinct subpopulations called CSCs [34] once the degree of genetic change reaches a tolerable threshold [111]. Since RNA expression correlates with gene copy numbers, scRNA‐seq enables the detection of chromosomal gains or losses by comparing transcriptional profiles from individual tumor cells to those of normal diploid references. Upregulated or downregulated gene expression across chromosomal regions reflects CNVs, which are then used to compute a chromosomal instability index (CII), quantitatively capturing CIN levels, distinguishing CIN^high^ from CIN^low^ in each cell across a tumor [74, 109]. Importantly, these CNV‐driven transcriptomic alterations are not merely by‐products of instability but serve as functional mediators of tumor cell reprogramming. CIN‐induced CNVs alter the expression of key oncogenic and stemness‐related genes, thereby enhancing genetic variability and promoting the emergence of CSCs. This occurs through chromosomal imbalance–driven stress responses that rewire transcriptional programs toward self‐renewal and pluripotency. Mechanistically, CIN can trigger de‐differentiation of non‐stem tumor cells via suppression of p53, amplification of cellular myelocytomatosis oncogene (c‐MYC), and aberrant activation of Wnt/β‐catenin signaling, all of which converge to sustain self‐renewal and pluripotency programs [58, 112]. Interestingly, the heterogeneous distribution of stem cell – associated markers across diploid and aneuploid tumor clones (Table 2), indicating that stem‐like phenotypes are maintained across genetically distinct karyotypic states rather than being restricted to a single ploidy class [113, 114, 115, 116]. Moreover, CIN promotes adaptive evolution within the tumor by enabling GSCs to acquire oncogenic alterations, including amplifications of EGFR, losses of PTEN, and mutations in TP53, which further exacerbate heterogeneity and drive aggressive tumor behavior [117] as shown in Table 3. Thus, single‐cell analyses reveal CIN fosters clonal selection, allowing specific subpopulations with advantageous karyotypic configurations to dominate and maintain intratumoral diversity [108, 109]. Once CSCs are established, they further contribute to tumor heterogeneity through asymmetrical division and lineage plasticity, enabling phenotypic switching under therapeutic pressure [56, 118].

**TABLE 2:** Expression of cell surface markers in CSCs with chromosomal aberrations.

Stemness marker	Evidence Linking Expression → Stemness / CSC Phenotype	Relevance to Genetic / Ploidy Variability
**CD56**	Expressed broadly, but not strictly CSC‐specific in GBM; not linked to stemness hierarchy [113].	Similar in both diploid and aneuploid clones in GBM [113].
**CD90**	Recognized as GSC marker with co‐expression with CD133; higher in high‐grade gliomas [114].	Not systematically studied for diploid vs aneuploid in main papers so far.
**CD29**	Often a broad stem/progenitor marker (integrin β1), used in combination panels [115].	No strong published ploidy correlation yet.
**A2B5**	Strong evidence of functional stemness (tumour initiation, neurosphere formation); varies across clones [168].	Shows variability between clones and associates with tumorigenic potential [113].
**CD133**	Widely studied CSC marker; enriched stemness features and associated outcomes; heterogeneous expression across tumors [116].	Differential expression between diploid and aneuploid clones [113].
**CD15**	Reported as a CSC marker in some GBM subsets; variable reliability [115].	Differential expression shown in clone studies [113].

Expression of glioblastoma stem‐cell (GSC)–associated surface markers is heterogeneous and does not uniformly correlate with chromosomal ploidy status. Multiple studies demonstrate that both diploid and aneuploid GBM clones can exhibit stem‐like phenotypes, with variable expression of markers such as CD133, CD15, A2B5, CD56, CD90, and CD29. This heterogeneity reflects underlying genetic divergence and cellular plasticity within tumors rather than a strict association between aneuploidy and stemness. Functional stem‐cell properties in GBM are therefore better defined by tumor‐initiating capacity and transcriptional programs than by individual surface markers alone.

**TABLE 3:** Molecular lesions associate with pathophysiology in GBM.

Pathology	IDH status	Subtypes	Molecular lesions
Grade IV glioblastoma	IDH mutant	Not specified	G‐CIMP, TP 53 mutation, ATRX mutation, CDKN2A/2B deletion, Chr. 10q loss.
	IDH wild type	RTK I	PDGFRA amplification, TRET promoter mutation, +7q/−10q genotype.
		RTK II	EGFR amplification, CDKN2A/2B deletion, PTEN mutation, TRET promoter mutation, +7q/−10q genotype.
		Mesenchymal	TERT promoter mutation, +7q/−10q genotype.
		H3F3A K27	HIST1H3B mutation, Activin A Receptor Type I (ACVR1) mutation, ATRX mutation, TP53 or Protein Phosphatase, Mg2+/Mn2+ Dependent 1D (PPM1D) mutation.
		H3F3A G34	H3F3A G34 mutation, ATRX mutation, TP53 mutation

Unlike IDH mutant type Grade IV glioblastoma, in case of IDH wild type GBM further classified into subtypes in the context of mutations in growth factor receptors of RTK family, mesenchymal phenotype and mutations in histone proteins. H3 Histone Family Member 3A (H3F3A).

In addition, CSCs and Epithelial–Mesenchymal transition (EMT)‐associated cells share markers such as CD44, CD133, and Aldehyde Dehydrogenase (ALDH) [119] supporting the EMT‐promoting role of CIN in cancer progression [120]. EMT contributes to cancer progression by enabling metastasis. Interestingly, EMT‐inducer Twist family bHLH transcription factor 1 (TWIST1) drives genomic instability in colorectal cancer, while CIN reciprocally alters genes regulating junctional proteins, reinforcing EMT and metastasis in ovarian tumors [112]. This creates a feed‐forward loop in which CIN and EMT cooperatively enhance malignancy and dissemination. While chromosomal abnormalities and EMT promote stemness, CIN‐driven alterations in protein expression have also been implicated in shaping the stem‐like state of astrocytoma IDH‐mutant, WHO grade 4, and IDH‐wildtype glioblastoma. Dysregulation of SRY‐Box Transcription Factor 2 (SOX2), Nestin, CD133, and AXL receptor tyrosine kinase (AXL), resulting from CIN, reinforces stemness and promotes therapy resistance [58, 109, 121]. In addition, CIN‐driven intrinsic cellular inflammatory response triggers Signal Transducer and Activator of Transcription 3 (STAT3), and NF‐κB pathways, which further augment self‐renewal and invasiveness [122].

Metabolic alterations driven by IDH mutations also contribute to GBM pathogenesis by maintaining CSCs as central drivers of aggressiveness and therapy resistance [123, 124]. The oncometabolite D‐2HG inhibits α‐ketoglutarate–dependent enzymes, altering epigenetic regulation via TET and histone demethylases, producing the hypermethylated Glioma‐CpG Island Methylator Phenotype (G‐CIMP phenotype) that impacts stem‐like transcriptional programs and differentiation [123, 125]. The G‐CIMP phenotype, defined by widespread CpG island hypermethylation, identifies a distinct molecular subset of gliomas characterized by epigenetic silencing of differentiation and lineage‐specific genes, thereby locking cells in a progenitor‐like state [126]. While G‐CIMP‐high gliomas—typically associated with IDH‐mutant backgrounds, exhibit reduced CIN and better prognosis, erosion of methylation (G‐CIMP‐low) parallels activation of stemness and cell‐cycle programs, contributing to increased plasticity, CIN, and aggressiveness reminiscent of IDH‐wild‐type GBM [127, 128]. Thus, the presence or loss of G‐CIMP methylation marks not only stratifies glioma subtypes but also mirrors the dynamic acquisition of stem‐cell‐like features that underpin therapeutic resistance and intratumoral heterogeneity. This collectively infers a mechanistic interplay between IDH mutational status and CIN‐associated attributes that sustains GSC plasticity and resilience, thereby reinforcing intra‐tumor heterogeneity [129] and driving therapeutic resistance.

## 10 Targeting CIN‐Inducing Mechanism for Therapeutic Intervention in Glioblastoma

The use of molecular characterization makes it easier to identify glioma and GBM subtypes, yet delivering a promising therapeutic regimen for GBM remains challenging. Despite multidisciplinary treatments, including surgery, chemotherapy, and radiotherapy, median survival for glioblastoma patients remains limited to 12–15 months. In most cases, GBM shows resistance to radiation and Temozolomide (TMZ), both of which are first‐line treatments following surgery [130]. TMZ resistance is influenced by MGMT activity, where MGMT overexpression repairs DNA by removing alkyl groups at the O6 position of guanine, counteracting TMZ‐induced cytotoxicity. Thus, methylation of the MGMT promoter can silence MGMT expression, leading to greater TMZ sensitivity [131]. However, not all GBM tumors follow this pattern. TMZ efficacy varies among MGMT‐unmethylated GBM tumors, as demonstrated in patient‐derived xenograft studies showing heterogeneous responses to temozolomide despite similar MGMT status [132]. Beyond promoter methylation, MGMT expression is regulated by multiple transcriptional and post‐transcriptional mechanisms. Several transcription factors, including Specificity Protein‐1 (Sp1), Nuclear Factor‐κB (NF‐κB), CCAAT/Enhancer‐Binding Protein (C/EBP), and Activator Protein‐1 (AP‐1), can modulate MGMT transcription, while microRNA‐mediated regulation, such as miR‐648‐dependent suppression of MGMT expression, further contributes to the heterogeneity of temozolomide resistance in glioblastoma [133, 134]. Therefore, in order to improve the therapeutic outcome in patients, targeted therapy against growth factor‐driven signaling pathways in GBM is incorporated. As a result, cell culture‐based studies regarding the association of growth factors, including EGF, Vascular Endothelial Growth Factor (VEGF), PDGF, HGF, FGF, and Insulin‐like Growth Factor (IGF‐1), with GBM pathogenesis attract the development of druggable‐targeted therapeutics. However, the outcome in patients with targeted therapies in clinical trials does not stand promising as observed in preclinical studies [135, 136]. Targeted therapies exploit cancer cells’ dependence on specific oncogenic drivers for survival; however, chromosomal instability can generate genomic diversity that enables cancer cells to bypass this oncogene dependency and develop resistance to targeted therapies [137]. This is consistent with the observation where only 10–20% of GBM patients respond to EGFR inhibitors due to heterogeneous EGFRvIII expression [138] or parallel signaling axis exerted from PDGFR amplification and mutations [139]. Therefore, CD133 or other GSC –specific markers are targeted for therapeutic intervention but the intratumoral heterogeneity of GSCs complicates biomarker identification and therapeutic targeting [140]. Mechanistic studies reveal that aneuploid cells activate the Rapidly Accelerated Fibrosarcoma‐ Mitogen‐activated protein kinase kinase‐Extracellular‐signal‐regulated kinase (RAF–MEK–ERK) pathway to overcome heightened DNA damage [141], while increased RNA and protein degradation counteract proteotoxic stress [142]. Chromosomal instability itself confers intrinsic multidrug resistance across diverse cancer types, as CIN‐positive cells display reduced drug sensitivity independent of proliferation rate or mutational profile. Moreover, transiently induced CIN accelerates the evolution of therapy resistance by generating karyotypic diversity that favors the emergence of adaptive clones [143], underscoring CIN as a dynamic driver of therapeutic failure [3]. These findings indicate that CIN and aneuploidy create distinct stress dependencies that can be therapeutically targeted.

Although chromosomal CIN–driven drug resistance is not fully categorized, targeting CIN‐promoting mechanisms remains promising. Microtubule‐destabilising agents such as vinca alkaloids, taxanes, and epothilones induce mitotic arrest [144] and induce mitotic‐catastrophe‐associated cell death [145], but GSCs resist these due to dormancy and ABC transporter overexpression [146]. Therefore, the requirement for alternative therapeutic approaches has emerged in the therapeutic landscape. In this context, several targeted therapeutics aimed at regulators of chromosomal instability and mitotic signaling are currently under investigation for glioblastoma, as summarized in Table 4. In addition, emerging small‐molecule inhibitors targeting key regulators of centrosome duplication, such as Nek2 and Plk4 kinases, have shown promise in preclinical models by selectively inducing apoptosis in cancer cells with amplified centrosomes while sparing normal cells [147]. Given NEK2's role in efflux‐driven resistance, its inhibition restores mitotic fidelity and may synergize with chemotherapy [148]. Thus, transcriptomic analyses demonstrate centrosome‐associated signatures define prognostic subtypes in gliomas [149]. In parallel, targeting spindle assembly checkpoint (SAC) regulators such as MPS1 can exploit mitotic vulnerabilities in glioblastoma; inhibition of MPS1 has been shown to enhance the efficacy of anticancer therapies and sensitize GBM cells to treatment modalities, including radiation [150]. Recent findings further emphasize the therapeutic promise of SAC modulation in gliomas. In glioblastoma, silencing BUB3, a core SAC component, enhances the anti‐proliferative effects of paclitaxel by promoting cellular senescence, suggesting that disrupting SAC fidelity can push tumor cells beyond their mitotic tolerance threshold [151]. Conversely, in colorectal cancer, Yin Yang 2 Transcription Factor (YY2)‐mediated upregulation of BUB3 hyperactivates the SAC, results in suppression of tumor progression [152], demonstrating that both weakening and hyperactivating SAC signaling can exert antitumor effects depending on the cellular context. Together, these studies highlight BUB3‐centered SAC modulation as a context‐dependent vulnerability that could be therapeutically exploited in gliomas to manipulate chromosomal instability and treatment response.

**TABLE 4:** Ongoing targeted therapeutics in clinical or translational development for glioblastoma targeting chromosomal instability (CIN) and mitotic regulatory pathways.

Target / Pathway	Drug / Inhibitor	Clinical Phase / Status	Mechanism Related to CIN	Consequence of Treatment (Observed / Proposed)
Aurora kinase A (AURKA) [169]	Alisertib (MLN8237)	Phase I/II trials and translational studies	Regulates centrosome maturation and spindle assembly during mitosis	Inhibition causes abnormal mitosis, polyploidy, mitotic catastrophe, apoptosis; enhances immune‐mediated tumor killing and may sensitize GBM cells to other therapies.
Aurora kinases (pan‑Aurora) [170]	Tozasertib (VX‑680)	Preclinical / early translational	Inhibits Aurora A/B kinases controlling chromosome alignment and cytokinesis	Induces polynucleation and apoptosis in GBM cells and suppresses tumor‐propagating cells when combined with radiation.
TTK / MPS1 kinase [171]	CFI‑402257; BAY‑1217389	Phase I–II trials in solid tumors; translational GBM studies	Inhibits spindle assembly checkpoint ensuring accurate chromosome segregation	Increased aneuploidy and apoptosis; and synergistic cytotoxicity with temozolomide in GBM cells.
CDK4/6–RB pathway [172]	AU3‐14	Phase I/II trials in GBM	Regulates G1‑S cell cycle transition linked to genomic stability	Cell cycle arrest, senescence, enhanced response to temozolomide.
EGFR signaling [135]	Erlotinib; Osimertinib	Multiple Phase II trials	EGFR amplification drives proliferation and replication stress contributing to genomic instability	Reduced tumor proliferation and signaling through PI3K/AKT and MAPK pathways.

The table summarizes selected targeted therapeutic agents currently under clinical or translational investigation for glioblastoma that modulate pathways associated with chromosomal instability (CIN), including mitotic kinases, spindle assembly checkpoint regulators, and oncogenic signaling pathways. The reported consequences of treatment are based on available preclinical or clinical evidences.

In addition, given the critical role of DNA damage response (DDR) pathways in maintaining genomic integrity, their therapeutic targeting has emerged as a promising strategy to exploit the inherent genomic instability of glioblastoma. For example, inhibitors of ATR, ATM, and PARP selectively impair the ability of glioblastoma cells to repair DNA damage, enhancing the efficacy of DNA‐damaging agents like radiation and TMZ [32]. Additionally, aneuploid cells depend on MAPK signaling for DNA repair balance [141], suggesting that combinatorial targeting of DDR and stress‐adaptive kinases could potentiate synthetic lethality. Recent drug delivery advances, including nanoparticle‐based systems, improve DDR‐targeting specificity and bioavailability [153]. Furthermore, new genome‐engineering tools such as KaryoCreate enable chromosome‐specific aneuploidy induction, providing mechanistic insights into CIN drivers and therapeutic vulnerabilities [154]. A comprehensive modeling study also harmonized CIN measurement metrics across mechanistic frameworks [155], offering a standardized foundation for assessing CIN‐targeted therapies.

Emerging evidence links cytoplasmic stress and nuclear integrity via a p62‐dependent mechanism that modulates micronuclear stability and CIN. p62 fine‐tunes micronuclear envelope rupture in response to oxidative stress, linking proteostasis and genome instability [62, 156]. This reinforces how oxidative or metabolic dysregulation feeds into CIN‐driven tumor evolution, bridging cytoplasmic stress signaling with genomic instability in GBM. From a therapeutic standpoint, modulating p62 activity provides two major strategies: inhibiting p62‐dependent autophagy could stabilize micronuclei and suppress CIN‐driven evolution, while transiently enhancing micronuclear rupture might augment immunogenicity through cGAS–STING activation [157]. Thus, selective p62 modulators or autophagy inhibitors such as hydroxychloroquine could be explored in rational combinations with DDR or MAPK inhibitors to prevent adaptive resistance [104, 158, 159]. These emerging directions position p62 as a mechanistically defined and druggable node linking stress response, chromosomal instability, and therapeutic sensitivity in GBM [160].

Along with the effort of designing small molecule inhibitors, CAR T‐cell therapy has emerged as a promising approach in glioblastoma treatment, offering the potential for highly specific and personalized immunotherapy [161]. Its advantages lie in the engineered T cells' ability to recognize and target Tumor‐Associated Antigens (TAAs) on glioblastoma cells, bypassing traditional immune evasion mechanisms [162]. Recent clinical advances, such as targeting antigens like EGFRvIII and Interleukin‐13 Receptor Subunit alpha‐2 (IL13Rα2), have demonstrated initial safety and efficacy in early‐phase trials, with some glioblastoma patients showing prolonged survival and tumor regression [163]. Hence, characterization of major cancer cell‐tolerant CIN that result in the expression alteration of cell surface marker plausibly broaden the scope of promising treatment through exploiting the advantage of engineered T cells' ability to recognize and TAAs on glioblastoma cells [162]. However, heterogeneity of antigen expression in glioblastoma, the risk of T‐cell exhaustion in the immunosuppressive microenvironment and the presence of the blood‐brain barrier raise a significant challenge in CAR‐T cell therapy [161, 162, 164]. Innovative strategies, including the development of multi‐antigen targeting CARs and the incorporation of safety switches to avoid auto‐immunity, are being explored in pan‐cancer models to enhance efficacy and minimize off‐tumor toxicity [162]; blood‐brain barrier penetration remains a challenge [165]. Nevertheless, integration of CAR‐T with CIN‐targeted therapies may enhance cytotoxic efficiency by exploiting genomic stress in tumor cells.

## 11 Summary

CIN is a central and under‐exploited axis of glioblastoma biology that not only accelerates tumor evolution but also creates clinically actionable liabilities. Growing evidence positions CIN—the continuous missegregation of whole chromosomes and structural rearrangements—as a major generator of intratumor karyotypic diversity that fuels clonal selection, therapeutic resistance, and the emergence or maintenance of GSC phenotypes. Despite advanced understanding on CIN‐promoting mechanisms, several knowledge gaps hinder the clinical translation of CIN research.

The field lacks standardized, clinically applicable CIN metrics, while various research‐grade approaches measure CIN through CNV burden, micronucleus frequency, or karyotype complexity, no consensus exists on a reproducible scoring system suitable for pathology or clinical trial use. Moreover, there is limited longitudinal and spatially resolved evidence linking CIN to the emergence of GSCs. Although several of the challenges discussed here, such as the lack of standardized CIN metrics and the need for longitudinal or spatially resolved datasets, have been recognized across the broader chromosomal instability literature, this review integrates these concepts specifically within the biological and clinical context of glioblastoma. By synthesizing mechanistic insights with emerging genomic, cytogenetic, and single‐cell technologies, we outline a disease‐focused framework for translating CIN from a descriptive feature of tumor evolution into a clinically actionable biomarker in GBM. Most existing data, which come from cross‐sectional studies, show that diploid and aneuploid clones often coexist with stemness‐associated markers; however, these studies do not clarify how CIN‐driven stemness evolves over time. Translationally validated assays such as single‐cell whole‐genome sequencing and spectral karyotyping remain costly and lack regulatory standardization, limiting their routine clinical deployment. Additionally, few clinical trials stratify patients by CIN status, leaving gaps in our understanding of which CIN signatures predict response to DDR inhibitors, mitotic stress‐inducing agents, centrosome‐targeting drugs, or immunotherapies leveraging micronucleus‐driven immunogenicity.

To address these challenges, an integrated, multi‐modal strategy is essential. Baseline tumor characterization at the time of surgery should include whole‐genome sequencing for global CNV profiling and single‐cell DNA or RNA sequencing to resolve clonal CNV architectures, stemness‐related transcriptional programs, and subclone compositions. Combined single‐cell genomic and transcriptomic approaches can precisely link karyotypic states to functional phenotypes. Spatially resolved techniques such as multiplexed Fluorescence In Situ Hybridization (FISH) and single‐molecule RNA FISH can validate key CNVs and stem markers within tissue context, while conventional cytogenetic or spectral karyotyping remains valuable for identifying chromothriptic and structurally complex events. Complementary biochemical assays—such as micronucleus detection, γH2AX and comet assays for DNA damage, immunofluorescence‐based centrosome counts, and cGAS–STING activation profiling—help annotate the functional consequences of CIN.

For longitudinal monitoring, plasma or cerebrospinal fluid‐derived cell‐free DNA analyzed through whole‐genome sequencing and targeted CNV panels can track dominant clones and emergent aneuploid subclones throughout treatment. Liquid biopsies combined with computational deconvolution could detect resistant karyotypes before radiographic progression, offering a window for timely therapeutic adaptation. Integrating a standardized CIN score, derived from CNV burden, structural complexity, and micronucleus index, into clinical pathology reports would allow stratification of patients into biomarker‐driven therapeutic arms—for instance, high‐CIN cases may benefit from DDR or mitotic stress‐based therapies, whereas low‐CIN, mutation‐dominated tumors might respond better to Receptor Tyrosine Kinase (RTK)‐targeted interventions.

Defining and applying CIN patterns in glioblastoma holds great promise for the development of personalized medicine. Distinct CIN architectures correspond to unique vulnerabilities. Tumors with extensive structural complexity and chromothripsis may show heightened sensitivity to PARP or ATR inhibitor combinations, while those with centrosome amplification might respond to HSET or PLK4 inhibitors that disrupt centrosome clustering. Dynamic monitoring of clonal CNV shifts could guide adaptive therapy design, enabling early intervention before the establishment of resistant populations. Furthermore, understanding the interplay between micronucleus burden, cGAS–STING activation, and the immune microenvironment could inform rational combinations of immunotherapies with agents that transiently augment cytosolic DNA to boost antitumor immunity without inducing immune exhaustion.

In conclusion, CIN represents both a challenge and an opportunity in glioblastoma management. Transitioning from descriptive observation to actionable biomarker status requires the standardization of CIN features, the adoption of scalable single‐cell and cytogenetic platforms to trace clonal evolution, and the integration of CIN metrics into trial designs and pathology workflows. Only by conceptualizing CIN as a quantifiable and manipulable biomarker—rather than as a manifestation of genomic disarray—can clinicians anticipate resistance, design adaptive regimens, and harness tumor evolution for therapeutic gain. The field must now prioritize longitudinal, lineage‐resolved studies that couple single‐cell lineage tracing, biochemical and cytogenetic assays, and liquid biopsy monitoring with interventional trials testing CIN‐guided strategies. Such integrated efforts will determine whether deciphering CIN patterns can transform glioblastoma's intrinsic genomic instability from a source of therapeutic frustration into a foundation for personalized and evolution‐informed medicine.

## Conflicts of Interest

The authors declare no conflicts of interest.

## Data Availability

The authors have nothing to report.
